# Fast Image Restoration for Spatially Varying Defocus Blur of Imaging Sensor

**DOI:** 10.3390/s150100880

**Published:** 2015-01-06

**Authors:** Hejin Cheong, Eunjung Chae, Eunsung Lee, Gwanghyun Jo, Joonki Paik

**Affiliations:** Department of Image, Chung-Ang University, 84 Heukseok-ro, Dongjak-gu, Seoul 156-756, Korea; E-Mails: hjcheong87@gmail.com (H.C.); cej8838@gmail.com (E.C.); lessel7@gmail.com (E.L.); jghjgh09@gmail.com (G.J.)

**Keywords:** image restoration, spatially varying blur, truncated constraint least-squares (TCLS) filter, point spread function estimation

## Abstract

This paper presents a fast adaptive image restoration method for removing spatially varying out-of-focus blur of a general imaging sensor. After estimating the parameters of space-variant point-spread-function (PSF) using the derivative in each uniformly blurred region, the proposed method performs spatially adaptive image restoration by selecting the optimal restoration filter according to the estimated blur parameters. Each restoration filter is implemented in the form of a combination of multiple FIR filters, which guarantees the fast image restoration without the need of iterative or recursive processing. Experimental results show that the proposed method outperforms existing space-invariant restoration methods in the sense of both objective and subjective performance measures. The proposed algorithm can be employed to a wide area of image restoration applications, such as mobile imaging devices, robot vision, and satellite image processing.

## 1. Introduction

Restoration of spatially varying out-of-focus blur is a fundamental problem in enhancing images acquired by various types of imaging sensors [1–4]. Despite the advances in various digital imaging techniques, restoration of spatially varying degradation is still a challenging issue because of the inherent limitation of imaging devices and the non-ideal image acquisition conditions. Although various image restoration algorithms have been proposed in the literature, most of them work under an unrealistic condition that, for example, there is only a single, space-invariant blur. High computational load and/or the iterative optimization are another burden on the practical employment of digital image restoration algorithms in a general imaging sensor.

As a well-known, basic image restoration algorithm, the constrained least-squares filter can remove space-invariant image degradation with suppressing noise amplification using an appropriately weighted smoothing constraint [5]. Kim *et al.* proposed a practically efficient restoration method by truncating coefficients of the constrained least-squares filter [6–8], which can be implemented in the form of the finite impulse response (FIR) filter. However, the original version of the truncated constrained least-squares (TCLS) filter cannot deal with a spatially varying blur.

For restoring a space-variant degradation, Kuthirummal estimated the out-of-focus blur using a computational camera [9], and Pertuz proposed a spatially adaptive restoration method by estimating a local blur from multiple images [10]. Kuthirummal's method requires a special optical part that is not easily implemented in a general camera, whereas Pertuz's method is not suitable for fast image restoration because of the use of multiple input images. Whyte and Xu estimated blurring components in small blocks and proposed the correspondingly adaptive restoration method using sparse representation, respectively, in [11,12]. Chan proposed a selective image restoration method by separating defocused areas [13]. Shen proposed a restoration method using ℓ_1_ and ℓ_2_ minimizations [14]. The above mentioned adaptive restoration algorithms commonly need enormous amount of computations and an indefinite processing time to estimate the degradation parameters and optimization-based adaptive restoration.

**Figure 1.**
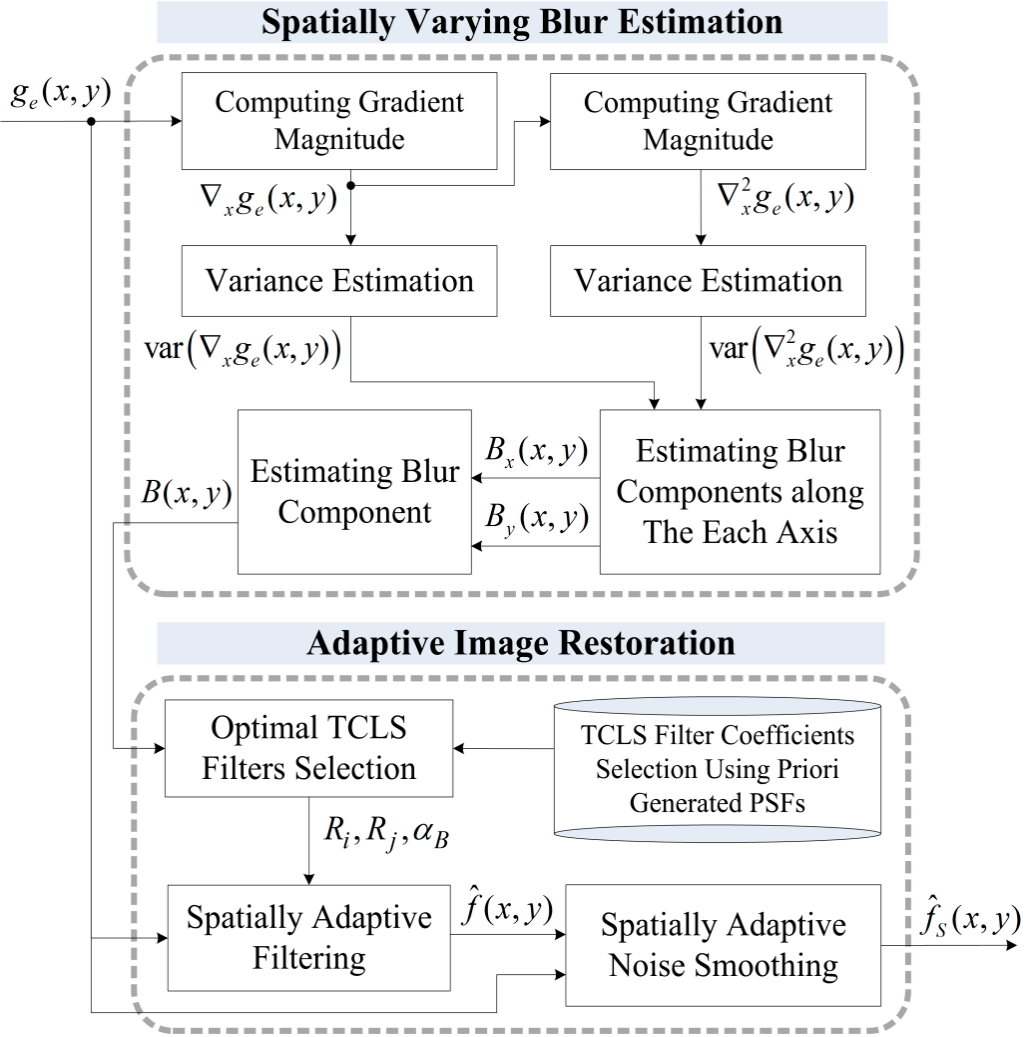
Block diagram of the proposed adaptive image restoration algorithm. (The derivative operations are shown in only the horizontal direction for simplicity but the real system computes both horizontal and vertical derivatives.).

In order to minimize the computational overhead of space-variant image restoration while preserving the restoration performance, this work assumes that spatially varying degradation can be approximated as multiple, region-wise space-invariant Gaussian kernels, and that the restoration process can be approximated in the form of an FIR filter. In this context, the proposed algorithm consists of two functional modules: (i) estimation of the spatially varying two-dimensional Gaussian point spread functions (PSFs) by analyzing the relationship between the first and second derivatives of the corresponding region and (ii) spatially adaptive image restoration using optimally selected TCLS filters as shown in Figure 1. The major advantage of the proposed method is the fast, robust restoration of spatially-varying defocus blur using the parametric model of the PSF and a computationally efficient FIR restoration filter. Although the real PSF of an optical lens is not necessarily Gaussian, the proposed parametric model is a good approximation of most optical lenses, which will be proved by the experiment.

Since spatially-varying image restoration is a fundamental problem in image filtering, enhancement, and restoration applications, there have been many researches in the literature. Some important techniques are summarized below with brief comparisons with the proposed work. Early efforts to enhance a satellite image assumed that the PSF varies because of a geometric transformation. In this context, Sawchuk proposed a space-variant image restoration method that performs a geometric transformation to make the blur kernel space-invariant, and then performed a space-invariant inverse filtering [15]. Flicker *et al.*, proposed a modified maximum-likelihood deconvolution method for astronomical adaptive optics images [16]. It is an improved version of anisoplanatic deconvolution using a space-varying kernel and Richardson-Lucy restoration. Flicker's method, however, differs from this work because of pre-required space-varying PSF information and optimization-based iterative restoration. Hajlaoui *et al.* proposed a spatially-varying restoration approach to enhance a satellite image acquired by a pushbroom-type sensor where the PSF is spatially varying in only one direction [17]. It uses wavelet decomposition with redundant wavelet frames to improve the restoration performance. However, MAP estimation and wavelet decomposition are not suitable to be implemented in the form of simple linear filtering. Hirsch *et al.* proposed a class of linear transformations called the Filter Flow for blind deconvolution of motion blur with noise, and they demonstrated the practical significance by showing experimental results on removing geometric rotation, atmospheric turbulence, and random motions [18]. Although this work has a common motivation of efficient space-variant deconvolution, PSF estimation of motion blur differs from that of the defocus blur.

This paper is organized as follows. In Section 2, a general space-variant image degradation process is approximated into a region-wise space-invariant model, which serves as a theoretical basis of the proposed space-adaptive image restoration. Sections 3 and 4, respectively, present the estimation of space-variant blur and the corresponding adaptive image restoration algorithms. After experimental results are given Section 5; Section 6 concludes the paper.

## 2. Region-Wise Space-Invariant Image Degradation Model

**Figure 2.**
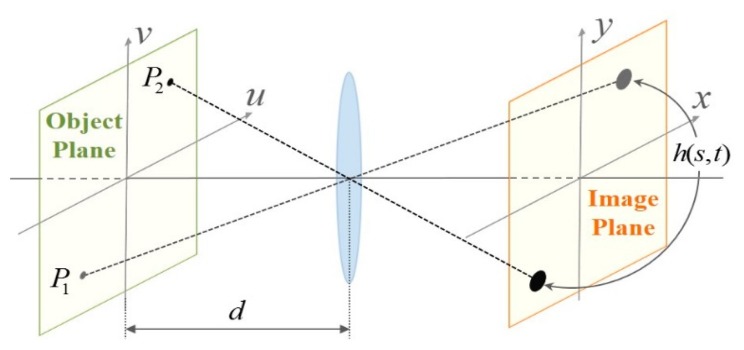
An image formation model of an object plane with distance *d* from the lens. The point spread function (PSF) *h*(*s*, *t*) is invariant throughout the image plane.

If spherical and chromatic aberrations of a lens are ignored, a point on the object plane generates a space-invariant point spread function (PSF) in the image plane as shown in Figure 2. The corresponding linear space-invariant image degradation model of the out-of-focus blur is expressed as a convolution sum as [19]

(1) $$g\left(x,y\right)=\sum_{s}\sum_{t}h\left(s,t\right)f\left(x-s,y-t\right)+\eta \left(x,y\right)$$

where *g*(*x*, *y*) represents the out-of-focus image, *f*(*x*, *y*) the virtually in-focused image assuming that the object plane is located at the in-focus position, η(*x*, *y*) the additive noise, and *h*(*s*, *t*) the space-invariant PSF.

**Figure 3.**
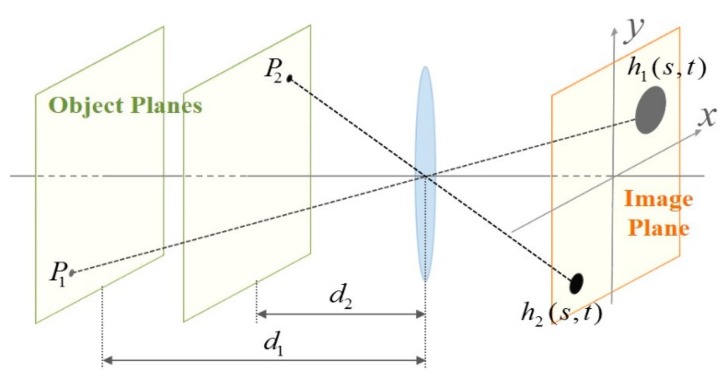
Image formation of two points with different distances from the lens, where *h*_1_(*s*, *t*) ≠ *h*_2_(*s*, *t*).

On the other hand, multiple objects with different distances from the lens generate different PSFs in the image plane as shown in Figure 3. In the corresponding space-variant image formation model, a PSF is determined by the distance of the object point from the lens. Since different object points are projected onto different locations in the image plane, a parametric representation of the Gaussian PSF is given as

(2) $$h\left(s,t;x,y\right)=h_{G}\left(s,t;\sigma_{xy}\right)=\frac{1}{2\pi \sigma_{xy}^{2}}\exp\left(\frac{-\left(s^{2}+t^{2}\right)}{2\sigma_{xy}^{2}}\right)$$

where *σ_xy_* varies with the location in the image plane. Equation (2) is a simplified version of a PSF for a single lens proposed in [20]. Elaboration of lens analysis and design is out of scope of the paper, and the proposed parametric method can represent other types of PSF with proper modifications. The corresponding space-variant image degradation model is given as

(3) $$g\left(x,y\right)=\sum_{s}\sum_{t}h_{G}\left(s,t;\sigma_{xy}\right)f\left(x-s,y-t\right)+\eta \left(x,y\right)$$

**Figure 4.**
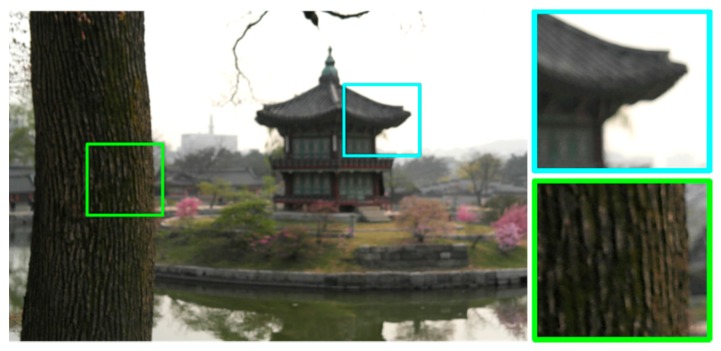
An outdoor image acquired by a digital single lens reflected (DSLR) camera. The gazebo and trunk of the tree are blurred by different PSFs.

Assuming that an image includes multiple objects (or regions) with different distances from the focus point and background, the space-variant model in Equation (3) can be approximated by a region-wise space-invariant version as

(4) $$g\left(x,y\right)=\sum_{i=1}^{K}A_{i}\left(x,y\right)\left\{\sum_{s}\sum_{t}h_{G}\left(s,t;\sigma_{i}\right)f\left(x-s,y-t\right)\right\}+\eta \left(x,y\right)$$

where the binary-valued function *A_i_*(*x*, *y*) is equal to unity only if a pixel at (*x*, *y*) is degraded by the *i*-th PSF *h_G_*(*s*, *t*; *σ_i_*), and *K* the number of different PSFs. The PSF *h_G_*(*s*, *t*; *σ_i_*) in Equation (4) is space-invariant in the region where *A_i_*(*x*, *y*) = 1, whereas *h_G_*(*s*, *t*; *σ_i_*) in Equation (3) is space-variant at each pixel. Figure 4 shows a real defocused image with multiple different PSFs. The tree and gazebo is blurred by different PSFs because they are located in different distances from the camera.

## 3. Blur Estimation Based on the Derivative Distribution

**Figure 5.**
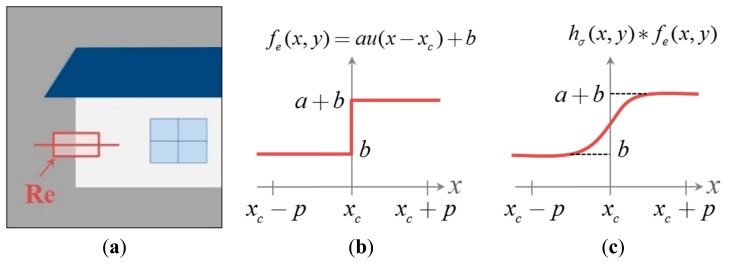
(**a**) A (2*p*+1) × (2*p*+1) region centered at (*x_c_*, *y_c_*), denoted as *R_e_*, containing only a single edge; (**b**) the ideal edge function along the horizontal line in *R_e_*; and (**c**) the convolution of the ideal edge function with a Gaussian blurring kernel.

In a sufficiently small region containing only a single, vertical edge as shown in Figure 5a, the ideal edge profile along the horizontal line can be expressed as

(5) $$f_{e}\left(x,y\right)=au\left(x-x_{c}\right)+b,\text{for}\;x_{c}-p\leq x\leq x_{c}+p\;\text{and}\;y_{c}-p\leq y\leq y_{c}+p$$

where *u*(*x*−*x_c_*) represents the unit step function, which is also called Heaviside function, shifted by *x_c_*, *a* the magnitude of the edge, and *b* the offset as shown in Figure 5b. If the region *R_e_* is out-focused by the Gaussian PSF that can be expressed as

(6) $$h_{\sigma }\left(x,y\right)=\frac{1}{2\pi \sigma^{2}}\exp\left(-\frac{x^{2}+y^{2}}{2\sigma^{2}}\right)$$

the correspondingly blurred edge function is given as

(7) $$g_{e}\left(x,y\right)=h_{\sigma }\left(x,y\right)*f_{e}\left(x,y\right)+\eta \left(x,y\right)=\left(h_{\sigma }*f_{\sigma }\right)\left(x,y\right)+\eta \left(x,y\right)$$

where η(*x*, *y*) represents the additive white Gaussian noise, such as $\eta \left(x,y\right)∼N\left(0,\sigma_{\eta }^{2}\right)$ . In the rest of this section, the shifting components *x_c_* and *y_c_* are omitted for simplicity.

In order to estimate the variance of the Gaussian PSF, an edge-based analysis approach is used. This approach is a parametrically modified version of two-dimensional isotropic PSF estimation using one-dimensional edge profile analysis [21]. Although one-dimensional edge can be in any direction, we derive the PSF estimation procedure in the horizontal direction, since any non-horizontal edges can be expressed as a rotated version of the horizontal edge. From the input degraded edge signal, a one-dimensional gradient of the blurred edge signal, that is the derivative of Equation (7) with respect to the *x*-axis without loss of generality, provides an important clue using the fundamental properties of the derivative of convolution and normal distribution as

(8) $$\begin{array}{ll} \nabla_{x}g_{e}\left(x,y\right) & =h_{\sigma }\left(x,y\right)*\nabla_{x}f_{e}\left(x,y\right)+\nabla_{x}\eta \left(x,y\right)\\ & =h_{\sigma }\left(x,y\right)*a\delta \left(x\right)+\nabla_{x}\eta \left(x,y\right)\\ & =a\underset{s}{\text{∑}}\underset{t}{\text{∑}}h_{\sigma }\left(s,t\right)\delta \left(x-s\right)+\nabla_{x}\eta \left(x,y\right)\\ & =a\underset{t}{\text{∑}}h_{\sigma }\left(x,t\right)+\eta \prime \left(x,y\right)\\ & =ah_{\sigma }\left(x,0\right)+\eta \prime \left(x,y\right) \end{array}$$

where $\eta \prime \left(x,y\right)=\nabla_{x}\eta \left(x,y\right)∼N\left(0,2\sigma_{\eta }^{2}\right)$ and the derivative operator ∇*_x_* can be replaced by a forward difference operator in the horizontal direction as

(9) $$\nabla_{x}f\left(x,y\right)=f\left(x+1,y\right)-f\left(x,y\right)$$

In one-dimensional case, the Gaussian parameter σ can be directly computed from the derivative equation by solving the Lambert W function [22]. However, it is not easy to solve the two-dimensional derivative equation given in Equation (8) for the Gaussian parameter.

Instead of directly solving Equation (8) for the Gaussian parameter σ, an approximate estimation of the amount of out-of-focus blur on the horizontal edge is given by the ratio of local variances of the first and second derivatives as

(10) $$\begin{array}{ll} B_{x}\left(x,y\right) & =\frac{\mathrm{var}\left\{\nabla_{x}g_{e}\left(x,y\right)\right\}}{\mathrm{var}\left\{\nabla_{x}^{2}g_{e}\left(x,y\right)\right\}}\\ & =\frac{E\left\{{\left(\nabla_{x}g_{e}\left(x,y\right)\right)}^{2}\right\}-E{\left\{\left(\nabla_{x}g_{e}\left(x,y\right)\right)\right\}}^{2}}{E\left\{{\left(\nabla_{x}^{2}g_{e}\left(x,y\right)\right)}^{2}\right\}-E{\left\{\left(\nabla_{x}^{2}g_{e}\left(x,y\right)\right)\right\}}^{2}}\\ & =\frac{E\left\{{\left(ah_{\sigma }\left(x,0\right)+\eta \prime \left(x,y\right)\right)}^{2}\right\}-E{\left\{\left(ah_{\sigma }\left(x,0\right)+\eta \prime \left(x,y\right)\right)\right\}}^{2}}{E\left\{{\left(-\frac{ax}{\sigma^{2}}h_{\sigma }\left(x,0\right)+\eta^{\prime \prime }\left(x,y\right)\right)}^{2}\right\}-E{\left\{\left(-\frac{ax}{\sigma^{2}}h_{\sigma }\left(x,0\right)+\eta^{\prime \prime }\left(x,y\right)\right)\right\}}^{2}}\\ & =\frac{a^{2}E\left\{h_{\sigma }{\left(x,0\right)}^{2}\right\}+2aE\left\{h_{\sigma }\left(x,0\right)\eta \prime \left(x,y\right)\right\}-a^{2}E{\left\{h_{\sigma }\left(x,0\right)\right\}}^{2}}{\frac{a^{2}}{\sigma^{4}}E\left\{x^{2}h_{\sigma }{\left(x,0\right)}^{2}\right\}-\frac{2a}{\sigma^{2}}E\left\{xh_{\sigma }\left(x,0\right)\eta^{\prime \prime }\left(x,y\right)\right\}-\frac{a^{2}}{\sigma^{4}}E{\left\{xh_{\sigma }\left(x,0\right)\right\}}^{2}} \end{array}$$

where var{·} represents the local variance and *E*{·} the local mean or average operators. The discrete approximation of the second order derivative operator $\nabla_{x}^{2}$ can be expressed as

(11) $$\nabla_{x}^{2}f\left(x,y\right)=\nabla_{x}\left\{\nabla_{x}f\left(x,y\right)\right\}$$

In Equation (10), both *E*{*h_σ_*(*x*, 0)η′(*x*, *y*)} and *E*{*xh_σ_*(*x*, 0)η″(*x*, *y*)} are close to 0 if we assume that both η′(*x*, *y*) and η″(*x*, *y*) are uncorrelated with *h_σ_*(*x*, 0). We have that, for (*x*, *y*) ∈ *R_e_*, where *R_e_* was defined in Figure 5a,

(12) $$\begin{array}{ll} B_{x}\left(x,y\right) & =\frac{a^{2}E\left\{h_{\sigma }{\left(x,0\right)}^{2}\right\}+2aE\left\{h_{\sigma }\left(x,0\right)\eta \prime \left(x,y\right)\right\}-a^{2}E{\left\{h_{\sigma }\left(x,0\right)\right\}}^{2}}{\frac{a^{2}}{\sigma^{4}}E\left\{x^{2}h_{\sigma }{\left(x,0\right)}^{2}\right\}-\frac{2a}{\sigma^{2}}E\left\{xh_{\sigma }\left(x,0\right)\eta^{\prime \prime }\left(x,y\right)\right\}-\frac{a^{2}}{\sigma^{4}}E{\left\{xh_{\sigma }\left(x,0\right)\right\}}^{2}}\\ & \approx \sigma^{4}\frac{E\left\{h_{\sigma }{\left(x,0\right)}^{2}\right\}-E{\left\{h_{\sigma }\left(x,0\right)\right\}}^{2}}{E\left\{x^{2}h_{\sigma }{\left(x,0\right)}^{2}\right\}-E{\left\{xh_{\sigma }\left(x,0\right)\right\}}^{2}}\\ & \propto \sigma^{4} \end{array}$$

which states that the amount of blur in the neighborhood of edge depends on the squared variance of the Gaussian PSF. The amount of blur on the vertical edge, *B_y_*(*x*, *y*), can also be computed in the same manner. The amount of the blur in an arbitrary direction is finally computed by combining the horizontal and vertical components as

(13) $$B\left(x,y\right)=\frac{B_{x}\left(x,y\right)B_{y}\left(x,y\right)}{\sqrt{B_{x}{\left(x,y\right)}^{2}+B_{y}{\left(x,y\right)}^{2}}}$$

The size of a local region *R_e_* is related to the range of an out-of-focus blur that is estimated using local statistics. If the size of a local region increases, a larger PSF can be estimated at the cost of potential mixture with other edges. In this work, *p* = 10 is used to estimate σ = 4.0 at maximum.

In flat areas, *B*(*x*, *y*) has a small value. It implies that both of a blurry object and a clear object have similar *B*(*x*, *y*) values in flat areas. However, the flat area does not need to be restored regardless of the amount of the defocus blur.

**Figure 6.**
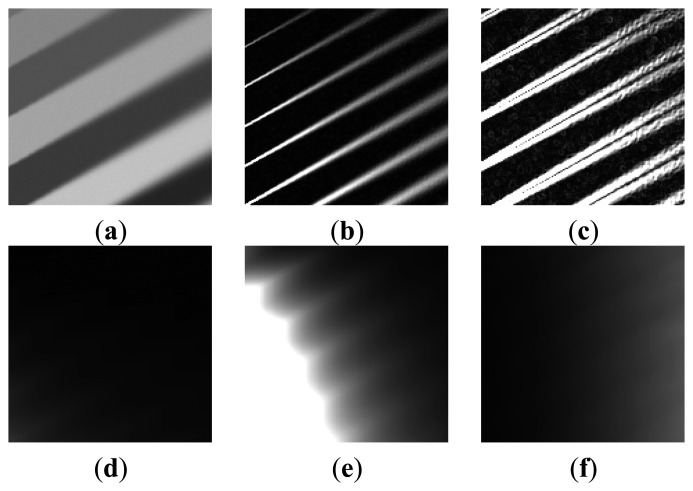
Computational procedure for estimating the blur component *B*(*x*, *y*)**:** (**a**) a synthetically degraded image by spatially varying blur; (**b**) the derivative of (a); (**c**) the second-order derivative of (a); (**d**) the local variance of (b); (**e**) the local variance of (c); and (**f**) the estimated blur component *B*(*x*, *y*)**.**

Figure 6 shows a step-by-step result of the proposed out-of-focus blur estimation process. Figure 6a show a synthetic image with gradually increasing amount of blur from left to right according to Equation (7). The variance of the Gaussian PSF changes from 0 to 2.5, and the variance of additive noise is 0.0001. Figure 6b,c, respectively, show the first- and second-order derivatives of the image. Figure 6d,e, respectively, show local variances of the first- and second-order derivatives. As shown in Figure 6f, the estimated amount of the blur *B*(*x*, *y*) follows the real amount of the blur regardless of the magnitude of edges.

## 4. Region-Adaptive Image Restoration Using Optimally Truncated Constrained Least-Squares Filters

Let *H*(*u*, *v*) represent the two-dimensional discrete Fourier transform (DFT) of the degradation function *h*(*x*, *y*) of the space-invariant degradation model, the corresponding constrained least-squares (CLS) restoration filter *R_CLS_*(*u*, *v*) is given as

(14) $$R_{CLS}\left(u,v\right)=\frac{H^{*}\left(u,v\right)}{{\left|H\left(u,v\right)\right|}^{2}+\lambda {\left|C\left(u,v\right)\right|}^{2}}$$

where *C*(*u*, *v*) represents a high-pass filter, and λ the regularization parameter for controlling the smoothness in the restored image [19]. In order to avoid the frequency domain processing that requires at least one additional frame memory, Kim has proposed the original version of truncated constrained least-squares (TCLS) filter [6], and also applied it to multi-focusing image restoration [8]. The TCLS filter is generated by truncating the spatial domain coefficients of the CLS filter using the raised cosine window. As a result, the TCLS filter can be realized in the form of a finite impulse response (FIR) filter in the spatial domain.

**Figure 7.**
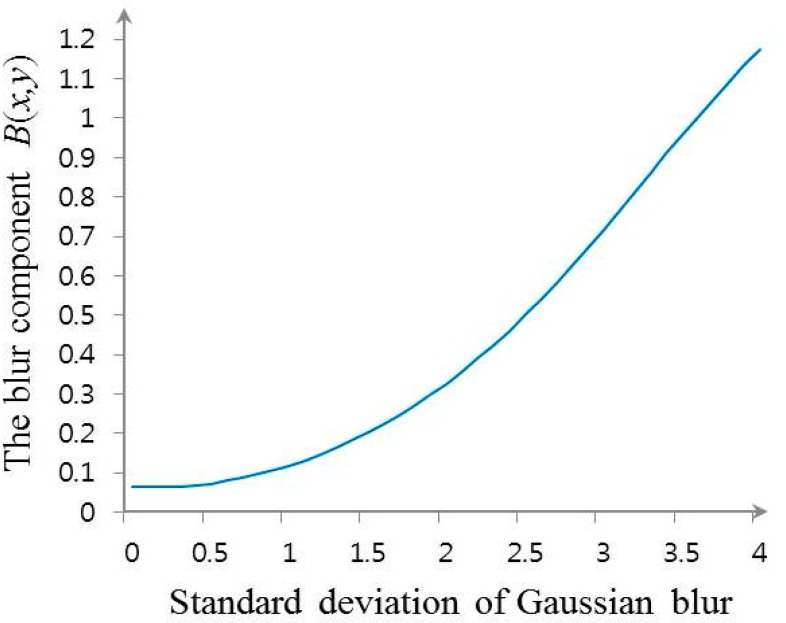
The blur size *B*(*x*, *y*) calculated with neighbors in the size of 21 × 21.

In order to design the TCLS restoration filter for the estimated blur size *B*(*x*, *y*), an arbitrary image is synthetically blurred by Gaussian function with variance σ^2^ to establish the relationship between *B*(*x*, *y*) and σ in a statistical manner. As shown in Figure 7, since *B*(*x*, *y*) and σ have the one-to-one correspondence, the optimum TCLS filter can be selected by calculating *B*(*x*, *y*) out of a set of *a priori* generated TCLS filters.

Estimated values of *B*(*x*, *y*) share adjacent intervals to select one of multiple TCLS filters for practically viable implementation. In this paper, the sharing step depends on the estimated values in a synthetically degraded images by Gaussian blurs that have various sizes according to Equation (1). The intervals of [0.08, 0.1), [0.1, 0.2), [0.2, 0.3), [0.3, 0.5), [0.5, 0.7), [0.7, 0.96), [0.96, 1.2), and [1.2, ∞) are used to determine the amount of blur according to the Gaussian functions with variances of 0.5, 1.0, 1.5, 2.0, 2.5, 3.0, 3.5, and 4.0, respectively. Although any number of intervals can be implemented in theory, we used nine intervals for tractable computational load.

In order to reduce the blocking artifacts caused by the quantization process, the proposed restoration method combines two optimum filters as

(15) $$\hat{f}\left(x,y\right)=\left\{\left(1-\alpha_{B}\left(x,y\right)\right)R_{i}\left(x,y\right)+\alpha_{B}\left(x,y\right)R_{j}\left(x,y\right)\right\}*g\left(x,y\right)$$

where *R_i_*(*x*, *y*) and *R_j_*(*x*, *y*) are two optimally selected TCLS filters, α*_B_*(*x*, *y*) the weighting factor for the appropriate fusion of restored images, and $\alpha_{B}\left(x,y\right)=\frac{B\left(x,y\right)-B_{i}}{B_{j}-B_{i}}$ , for 0 ≤ α*_B_*(*x*, *y*) ≤ 1. *B_i_* and *B_j_*, respectively, represent the minimum and maximum values of the section that *B*(*x*, *y*) belongs to. To reduce common restoration artifacts, such as noise clustering, ringing, and overshoot near edges, the spatially adaptive noise-smoothing algorithm [6,8] can also be used on the necessity basis.

**Figure 8.**
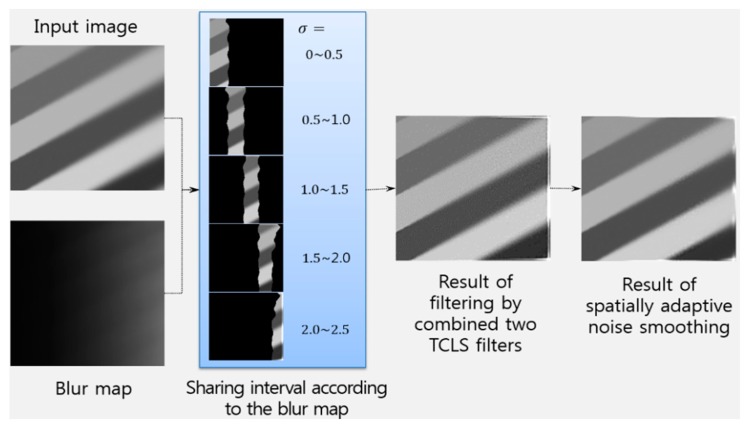
Procedure of the proposed region-adaptive image restoration algorithm.

Figure 8 shows an example for of the proposed region-adaptive image restoration algorithm. A spatially variant defocused input image is segmented for selecting optimal TCLS filters according to the blur map defined in Equation (10). The blurred image can be restored using Equation (15) together with the spatially adaptive noise smoothing algorithm.

The process of the proposed image restoration is shown as Algorithm 1. The proposed blur estimation algorithm corresponds to lines 1–2 in Algorithm 1 and the proposed region-adaptive restoration algorithm corresponds to lines 3–5.

**Algorithm 1** The proposed image restoration
**Inputs:** an input image *g_e_*(*x*, *y*), a set of TCLS filters, a table to share interval for selecting optimal filters.1. Estimate the amounts of blur as 
$B_{x}\left(x,y\right)=\frac{\mathrm{var}\left\{\nabla_{x}g_{e}\left(x,y\right)\right\}}{\mathrm{var}\left\{\nabla_{x}^{2}g_{e}\left(x,y\right)\right\}}$ and 
$B_{y}\left(x,y\right)=\frac{\mathrm{var}\left\{\nabla_{y}g_{e}\left(x,y\right)\right\}}{\mathrm{var}\left\{\nabla_{y}^{2}g_{e}\left(x,y\right)\right\}}$.2. Calculate the blur map *B*(*x*, *y*) according to 
$B\left(x,y\right)=\frac{B_{x}\left(x,y\right)B_{y}\left(x,y\right)}{\sqrt{B_{x}{\left(x,y\right)}^{2}+B_{y}{\left(x,y\right)}^{2}}}$.3. Select two optimal TCLS filters *R_i_*(*x*, *y*) and *R_i_*(*x*, *y*) using the blur map and the input table.4. Restore adaptively the input image by *f̂*(*x*, *y*) = {(1 − α*_B_*(*x*, *y*)) *R_i_*(*x*, *y*) + α*_B_*(*x*, *y*) *R_j_*(*x*, *y*)} * *g*(*x*, *y*).5. Apply the spatially adaptive noise smoothing algorithm.**Output**: a space adaptively restored image.


## 5. Experimental Results

For evaluating the performance of the proposed algorithm, Three sets of test images including a synthetic image, standard images of size 768 × 512, and outdoor images of size 1024 × 768 acquired by using a digital single lens reflected (DSLR) camera were tested using the peak-to-peak signal-to-noise ratio (PSNR), mean structural similarity (MSSIM) [23], and the CPU processing time in seconds on a PC equipped with a 3.40 GHz CPU and 16 GB RAM.

**Figure 9.**
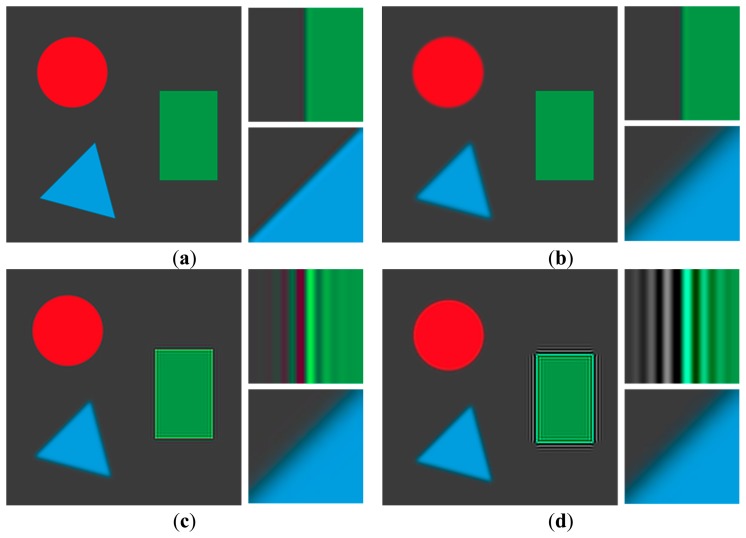

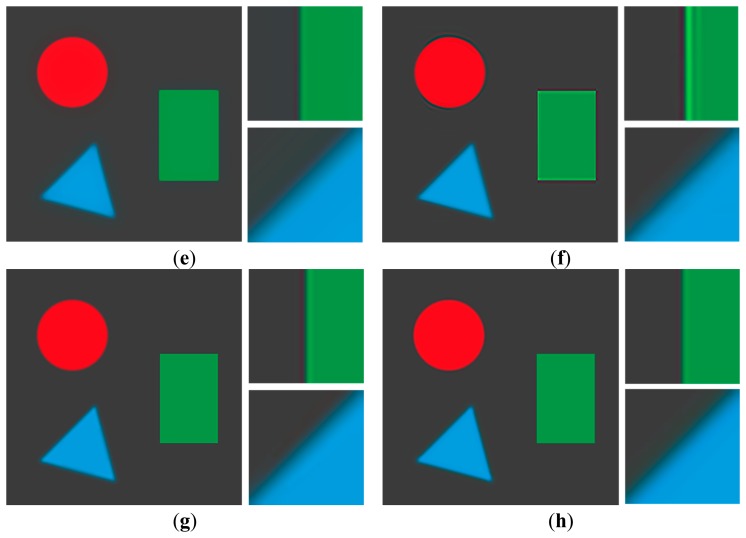
Comparison of different restoration algorithms using a synthetic image; (**a**) the input image; (**b**) the degraded image by spatially varying blur; (**c**) the restored image by the constrained least squares (CLS) filter (peak-to-peak signal-to-noise ratio (PSNR) = 33.13, mean structural similarity (MSSIM) = 0.94); (**d**) the restored image by the Dong's method [24] (PSNR = 26.68, MSSIM = 0.84); (**e**) the restored image by the Yang's method [25] (PSNR = 36.44, MSSIM = 0.97); (**f**) the restored image by the Xu's method [12] (PSNR = 29.62, MSSIM = 0.93); (**g**) the restored image by the Shen's method [14] (PSNR = 36.88, MSSIM = 0.98); and (**h**) the restored image by the proposed method (PSNR = 39.19, MSSIM = 0.98).

**Figure 10.**
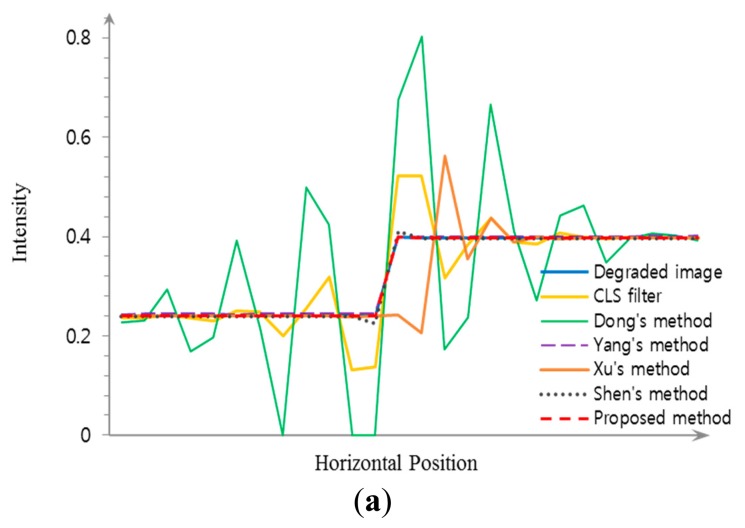

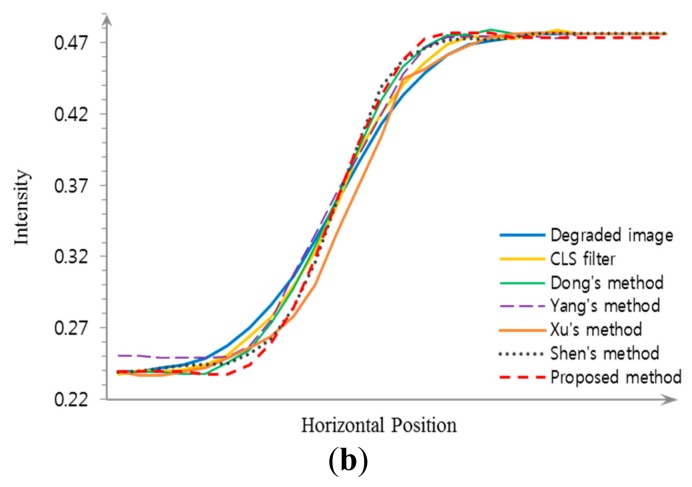
Comparison of different restoration algorithms using the ideal step function; (**a**) the step responses of edge around the rectangle in Figure 9b; (**b**) the step responses of edge around the triangle in Figure 9b.

Experimental results using a synthetic image are shown in Figure 9. As shown Figure 9c,d, the CLS filter and Dong's method result in significant ringing artifacts because of the mismatch between the real blur and the restoration filter. Although Yang's method that minimizes the total variation can better remove the defocus blur artifact with suppressed ringing as shown in Figure 9e than the CLS filter and Dong's method, it cannot avoid distortions in high frequency regions, such as corners and ridges. Its iterative computational structure is another burden to be implemented in a commercial digital camera. The result of Xu's method include ringing artifact as shown in Figure 9f. Since Xu's method globally estimates a blur kernel in the entire input image and then adjusts the blur kernel for each divided region, it is not easy to accurately measure the spatially varying defocus blur that may continuously changes throughout the image. Figure 9g,h show that both Shen's and the proposed restoration methods can successfully restore the spatially varying defocus blur without ringing artifacts. Although both methods perform restoration based on the blur map, Shen's method is strongly influenced by noise because it uses only maximum and minimum values within a local window for generating the blur map, which results in artifacts at boundaries with discontinuity of the blur map. On the other hand, the proposed method is more robust to noise since it uses the ratio of variances of the first and second derivatives in the corresponding region. The precisely estimated blur map by the proposed method results in less ringing artifact with the highest PSNR and MSSIM values. Figure 10a,b respectively show the averaged step responses of various restoration methods used in Figure 9. The restored step response using the proposed method is the most similar to that of the input image with minimum undesired artifacts.

In addition, the propose method is faster than other restoration methods. More specifically, for an image with *M* pixels, computational complexities of the proposed defocus blur estimation and spatially adaptive restoration algorithms are both equal to O(*M*). Thus, computational complexity of the proposed algorithm is equal to O(*M*). The computational complexities of Dong's and Yang's methods are both equal to O(*NM*), where *N* represents the number of iterations. In Shen's method, the computational complexity of the blur estimation image restoration algorithms are respectively equal to O(*M*) and O(*NM*) because of using ℓ_1_ − ℓ_2_ optimizations with *N* iterations. The computational complexity of Xu's method is also equal to O(*NM*) because of its iterative structure.

**Figure 11.**
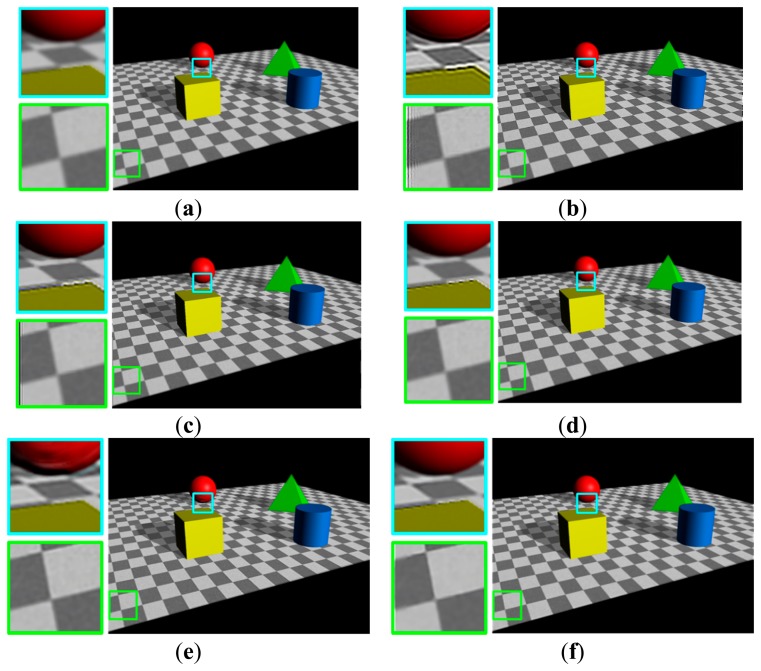
Comparison of different restoration algorithms using another synthetic image generated by a commercial three-dimensional computer graphics software; (**a**) the input image; (**b**) the restored image by the Dong's method [24] (PSNR = 22.62, MSSIM = 0.94); (**c**) the restored image by the Yang's method [25] (PSNR = 29.00, MSSIM = 0.98); (**d**) the restored image by the Xu's method [12] (PSNR = 26.17, MSSIM = 0.96); (**e**) the restored image by the Shen's method [14] (PSNR = 30.21, MSSIM = 0.98); and (**f**) the restored image by the proposed method (PSNR = 35.44, MSSIM = 0.99).

Figure 11 shows the results in another synthetic image which was generated using a commercial three-dimensional computer graphics software. Distances of the cylinder, cube, cone, and sphere to the camera lens is respectively equal to 15.5, 17, 24, and 25.5 cm with the in-focus distance of 17 cm. The proposed restoration method shows the best restored result in the sense of PSNR and MSSIM values and restores the spatially varying blur with minimum artifacts.

**Figure 12.**
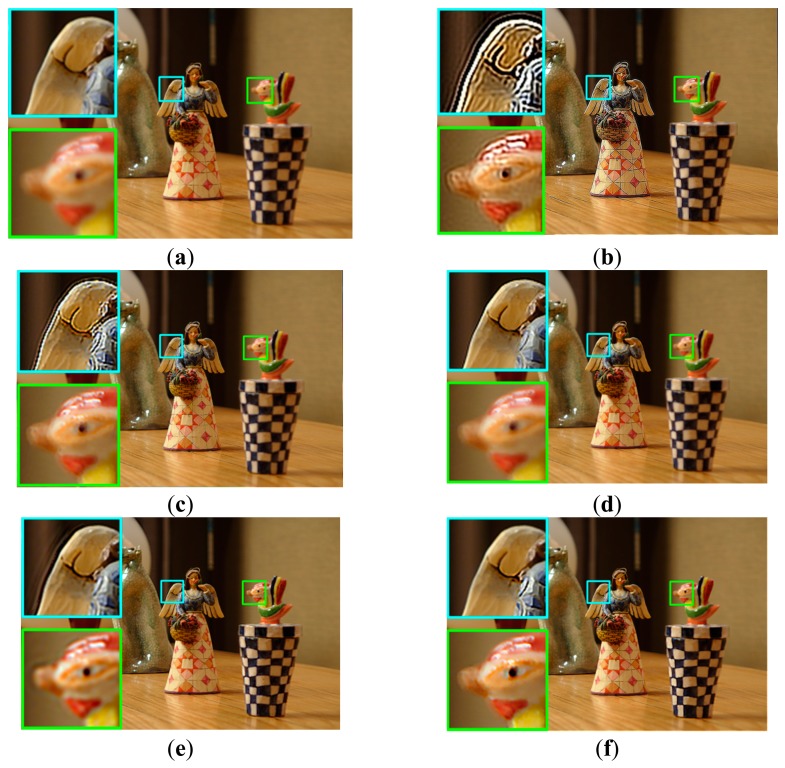
Comparison of different restoration algorithms using a real image with multiple objects with different distances from the camera; (**a**) the input image; (**b**) the restored image by the Dong's method [24]; (**c**) the restored image by the Yang's method [25]; (**d**) the restored image by the Xu's method [12]; (**e**) the restored image by the Shen's method [14]; and (**f**) the restored image by the proposed method.

**Figure 13.**
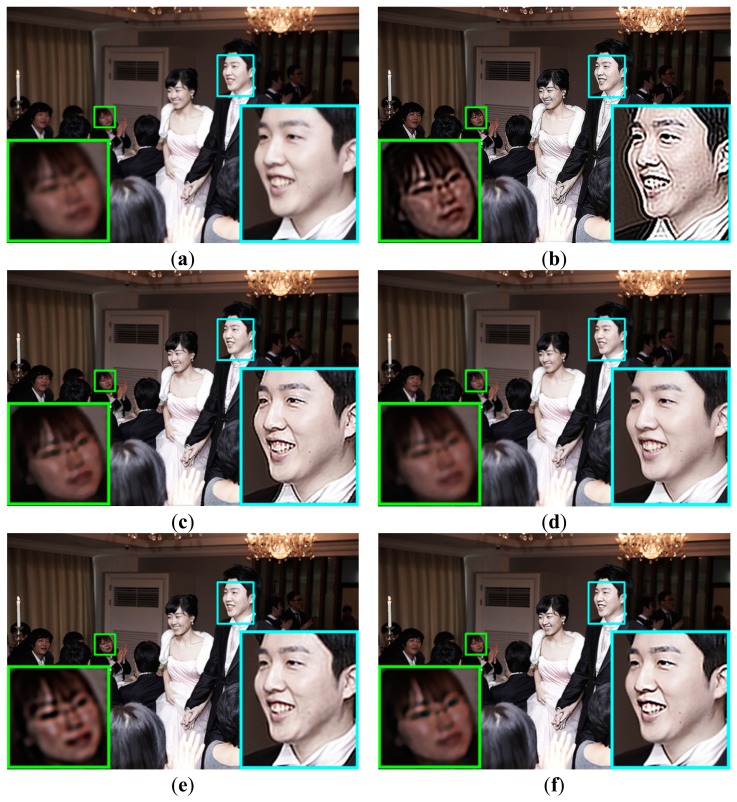
Comparison of different restoration algorithms using another real image; (**a**) the input image; (**b**) the restored image by the Dong's method [24]; (**c**) the restored image by the Yang's method [25]; (**d**) the restored image by the Xu's method [12]; (**e**) the restored image by the Shen's method [14]; and (**f**) the restored image by the proposed method.

Experimental results of the proposed method for naturally blurred images acquired by a DSLR camera are shown in Figures 12 and 13. Shen's method cannot avoid ringing artifacts at boundaries with discontinuity of the blur map. However, as shown in Figures 12d and 13d, the proposed restoration method successfully removes the spatially varying blur with minimized artifacts.

Table 1 summarizes PSNR/MSSIM values and the CPU processing time in seconds of Dong's, Yang's, Xu's, Shen's, and the proposed methods. To analyze image quality, each image was degraded using different Gaussian blurs where σ increases from the outside to the center of the image as shown in thumbnail figures of Table 1, and the images are restored using different methods. Dong's method shows lower PSNR and MSSIM values compared with other methods because of the magnified ringing artifacts. When noise variance is large, Yang's method is effective because it can control the noise by minimizing the total variation of the image, whereas its performance is not acceptable when the noise is negligible. Xu's method also shows low PSNR and MSSIM values due to inappropriate blur estimation for the spatially varying defocus blur. Although Shen's method gives almost similar PSNR and MSSIM values compared with the proposed method, its performance is limited in the neighborhood of the discontinuity in the blur map. The proposed method produces the best restored result in the sense of both PSNR and MSSIM values. In addition, the proposed algorithm can be implemented in the form of an FIR filter.

## 6. Conclusions

In order to solve the long-term unsolved digital multi-focusing problem in digital imaging technology, a region-wise linear approximation of the general space-variant image degradation model is presented. The proposed image degradation model can cover from the space-invariant to pixel-wise adaptive image restoration by adjusting the size of the region. Gaussian approximation of the point spread function (PSF) of an optical system enables parametric estimation of the blur parameter, which is an important factor for the potential application of wide variety of imaging sensors. Based on the region-wise space-invariant image degradation model, a novel Gaussian parameter estimation method is proposed by analyzing the relationship between the variance of a Gaussian PSF and the first and second derivatives of local edges. Since the proposed estimation method uses a set of *a priori* generated real out-of-focused images in the statistical manner, the estimation process is stable and free from amplification of the estimation error due to the ill-posedness of derivative operations.

The restoration process is implemented in the form of a finite impulse response (FIR) filter, which can be embedded in a typical image signal processor (ISP) of a digital imaging devices without using an additional hardware such as a frame memory. Although the truncation of the filter coefficients may degrade the performance of restoration in a certain degree, the region-based processing minimizes the degradation, which was proved by experimental results using a set of real photographs.

The major contribution of this work is that the multi-focusing problem is decomposed into two separate sub-problems: (i) estimation of the PSF and (ii) spatially-adaptive image restoration. Since neither perfect estimation of the PSF nor ideal restoration is possible in practice, in a combined approach, such as blind deconvolution, the mixed errors from the PSF estimation and restoration cannot be removed by an analytic method. On the other hand, in the proposed approach, PSF estimation can be improved by extending the Gaussian model to more realistic one without affecting the restoration process. In the same manner, the restoration process can be either improved or replaced with any advanced one without affecting the PSF estimation process. For example, Wei *et al.* proposed a matrix source coding algorithm for efficiently computing space-varying convolution, and demonstrated its performance using the space-variant image restoration results [26]. Since the proposed work completely decouples the PSF estimation and restoration sub-problems, Wei's convolution method can be applied to the restoration process to improve the performance.

## Figures and Tables

**Table 1.** Comparison of restoration performance and processing time for three test images, where the center region is blurred by Gaussian with σ = 3.0, and the peripheral region is blurred with σ = 0.8.

**Input Image**	**Restoration Method**	**PSNR [dB]**			**MSSIM**			**Average Processing Time [second]**

		**σ**_η_ **= 0.001**	**0.0001**	**0.00001**	**σ**_η_ **= 0.001**	**0.0001**	**0.00001**	
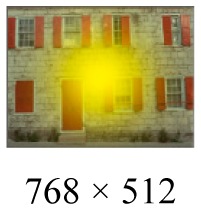	Dong [24]	18.81	20.43	20.59	0.69	0.79	0.81	309.5804
	Yang [25]	22.92	23.29	22.94	0.78	0.87	0.91	5.5511
	Xu [12]	18.77	22.01	22.23	0.75	0.84	0.84	1736.3780
	Shen [14]	23.81	25.62	25.76	0.82	0.90	0.91	66.1389
	Proposed	**24.43**	**26.75**	**27.47**	**0.83**	**0.91**	**0.92**	**0.6401**

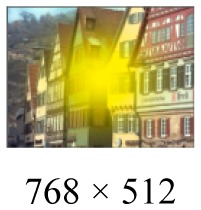	Dong [24]	17.96	19.46	19.57	0.74	0.83	0.85	315.0760
	Yang [25]	22.13	22.64	22.03	0.83	0.89	0.93	5.8107
	Xu [12]	15.02	18.00	18.10	0.66	078	0.79	1592.6384
	Shen [14]	22.31	23.34	23.35	0.86	0.91	0.92	68.7075
	Proposed	**22.38**	**25.21**	**25.84**	**0.87**	**0.93**	**0.94**	**0.5869**

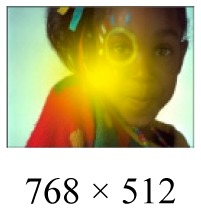	Dong [24]	24.57	28.87	29.88	0.60	0.86	0.93	287.5568
	Yang [25]	25.40	25.88	25.77	0.65	0.79	0.88	4.8221
	Xu [12]	20.29	29.57	30.17	0.73	0.91	0.94	2025.9904
	Shen [14]	26.55	**31.72**	31.55	0.79	0.93	0.94	72.4971
	Proposed	**28.36**	31.48	**32.78**	**0.81**	**0.94**	**0.96**	**0.5938**
